# Molecular data storage with zero synthetic effort and simple read-out

**DOI:** 10.1038/s41598-022-18108-9

**Published:** 2022-08-16

**Authors:** Philipp Bohn, Maximilian P. Weisel, Jonas Wolfs, Michael A. R. Meier

**Affiliations:** 1 Laboratory of Applied Chemistry, Institute of Organic Chemistry (IOC), Karlsruhe Institute of Technology (KIT), Straße am Forum 7, 76131 Karlsruhe, Germany; 2 Institute of Biological and Chemical Systems – Functional Molecular Systems (IBCS-FMS), Karlsruhe Institute of Technology (KIT), Hermann-von-Helmholtz-Platz 1, 76344 Eggenstein-Leopoldshafen, Germany

**Keywords:** Analytical chemistry, Cheminformatics

## Abstract

Compound mixtures represent an alternative, additional approach to DNA and synthetic sequence-defined macromolecules in the field of non-conventional molecular data storage, which may be useful depending on the target application. Here, we report a fast and efficient method for information storage in molecular mixtures by the direct use of commercially available chemicals and thus, zero synthetic steps need to be performed. As a proof of principle, a binary coding language is used for encoding words in ASCII or black and white pixels of a bitmap. This way, we stored a 25 × 25-pixel QR code (625 bits) and a picture of the same size. Decoding of the written information is achieved via spectroscopic (^1^H NMR) or chromatographic (gas chromatography) analysis. In addition, for a faster and automated read-out of the data, we developed a decoding software, which also orders the data sets according to an internal “ordering” standard. Molecular keys or anticounterfeiting are possible areas of application for information-containing compound mixtures.

## Introduction

The demand for non-conventional data storage solutions is increasing due to digitization and the enormous growth in data volumes worldwide. While the total amount of data globally was around 5 ZB in 2011, it reached 79 ZB in 2021 and is growing exponentially and is expected to reach 181 ZB in 2025^1^. As the data carrier of life, DNA has come increasingly into focus as a possible alternative in recent years^2–6^. The data density of DNA is higher than in magnetic tapes, the read-out is well investigated via sequencing approaches^7^ and it can store information for thousands of years^8^. In the context of this manuscript, the term "molecular storage" refers to the storage of information at a molecular level using defined single molecules, which could additionally be used in the form of compound mixtures.

Inspired by DNA, an increasing and continuing focus on methods for the preparation of synthetic sequence-defined molecules over the last ten years is observed^9–25^. Such unique macromolecules have lately gained interest in life and material science, e.g. as data storage devices^16^. While DNA is limited to the four information-containing nucleobases and thus long sequences are needed to store large amounts of information, the building blocks for coding in synthetic molecules are more diverse. In this context, Lutz et al. have presented the potential of sequence-defined poly(phosphodiester)s^26–29^, oligo(triazole amide)s^30,31^, oligo(alkoxyamine amide)s^32,33^, oligourethanes^34,35^ and oligo(alkoxyamine phosphodiester)s^36,37^ as so-called digital polymers. For the latter two substance classes, decoding and imaging from a surface via DESI was shown recently^38^. Information-containing oligomers, obtained by a thia-maleimide Michael coupling and read-out using MALDI-TOF MS/MS, were reported by Zhang and coworkers^39,40^. Kéki focused on an alcohol-isocyanate click approach for the synthesis of encoded polyethylene glycol^41^. In 2021, Yao et al. published the storage of data in peptide sequences^42^, and Anslyn and coworkers in self-immolative sequence defined urethanes^43^. These approaches are all based on using two monomer units, resulting in a binary code along the sequence. In order to store larger amounts of data, long sequences have to be synthesized, which is time-consuming and bears difficulties in terms of the read-out via tandem MS. Addressing the first point, automatic synthesis was used, reducing the reaction time and allowing an easy parallelization^44–47^. A recent example was shown by the group of Kim using semiautomated synthesis of poly(L-lactic-*co*-glycolic acid)s (PLGAs) and storage of 896 bits in 14 compounds (64-mers)^48^. Another approach is the shortening of the chain length by increasing the data density per monomer unit. Research in the direction of multifunctional sidechains has been reported by Ding et al. for polytriazoles^49,50^, by Barner-Kowollik via a synthesis based on photoligation^51^ and an approach by Du Prez based on thiolactone chemistry, presenting the en- and decoding of a 33 × 33-pixel QR code (1089 bits) with 71 oligomers^47^. Further methods to increase the complexity of the repeating units rely on dual side chain^52,53^, backbone and side chain^54,55^, dual side chain and backbone^56^ or dual side chain and dual backbone^57^ control and were successfully decoded applying tandem MS analysis. The read-out of mixtures of sequence defined oligomers (three hexamers, up to 64 bits in total), avoiding the synthesis of longer sequences, was recently reported by our working group^58^.

To further simplify the procedures, small molecules can be used for the storage of data as well. Highly complex small molecules, i.e. made by multicomponent reactions, exhibit a high data storage density and can be used, e.g., as a steganographic key for secret communication^59,60^. In addition, the writing and read-out of a 0.88 megapixel drawing of Pablo Picasso has been demonstrated by Rosenstein et al. using up to 1536 unique molecular mixtures of up to 575 different compounds with an accuracy of 97.57%^61^. Whereas all methods described so far are based on the read-out via fragmenting mass analysis, in the latter case only the presence or absence of the molecular mass within the corresponding mixture was decisive for the transmission of information. Thus, each molecule represents one bit of information. The storage of data > 100 kbits using synthetic mixtures of metabolites was demonstrated by the same group. Mixtures of up to 36 unique compounds were spotted on a steel plate and decoding was performed with accuracy > 99% via MALDI-MS^62^. The same strategy was used by Whitesides using mixtures of commercially available small oligopeptides analyzed by MALDI-MS^63^. In total, 400 kbit of information was written in mixtures of up to 32 compounds with 8 bits/s on a gold surface and retranslated with 20 bit/s with > 99% accuracy. The “Principles of Information Storage in Small-Molecule Mixtures” is explained in detail by Rosenstein et al.^64^. They theoretically point out the immense storage capacity and density of small-molecule mixtures, underlined by experimental demonstrations^61^. It is also addressed, that the read-out is not mandatorily restricted to MS or tandem MS, but can also be performed utilizing spectroscopic or chromatographic analysis^61^. Mixtures of fluorescent dyes for writing approximately 400 kbits of data in a binary code at a rate of 128 bits/s on a surface, and decoding these at a rate of 469 bits/s with > 99% accuracy via a confocal microscope, were demonstrated^65^. Another example in this context using Raman scattering of alkynes was described by Gao and coworkers^66^. A binary code was encoded in mixtures of up to 22 aromatic compounds by Keinan et al. using their own coding language and making use of specific chemical shifts and concentration dependent integral values in ^1^H NMR spectroscopy^67^. A similar approach is used in NMR photography to draw images with molecules based on their chemical shifts^68^.

The ever-increasing amount of information encoded in either sequence defined macromolecules or molecular mixtures entails the handling of ever-larger data sets. Thus, writing the data and the subsequent manual decoding reach their limits. For writing, increasingly automated synthesis and chemical printers are used, and software is being developed for processing the amount of data and reading out the original information^47,58,69^.

In this work, we show the data storage in molecule mixtures of commercially available chemicals, which enabled a fast and efficient preparation of the individual samples, if compared to synthetic approaches. The subsequent decoding was performed applying basic analytical tools (^1^H NMR spectroscopy and gas chromatography (GC)). We made use of a simple comparison approach, where the absence and the presence of a molecule, and its position in the respective spectrum or chromatogram, are used as binary information to carry either the information of an ASCII code or the black and white pixel of a bitmap. We further demonstrate a smart solution for the ordering issue, when handling more than one coding sample, by making use of the linear dependence of the integral on the peak concentration (GC). Furthermore, a software for decoding information from the compound mixtures analyzed by GC is introduced and showed a reliable readout for two 25 × 25-pixel bitmaps.

## Results

### Molecular data storage using NMR spectroscopy

**Figure 1:**
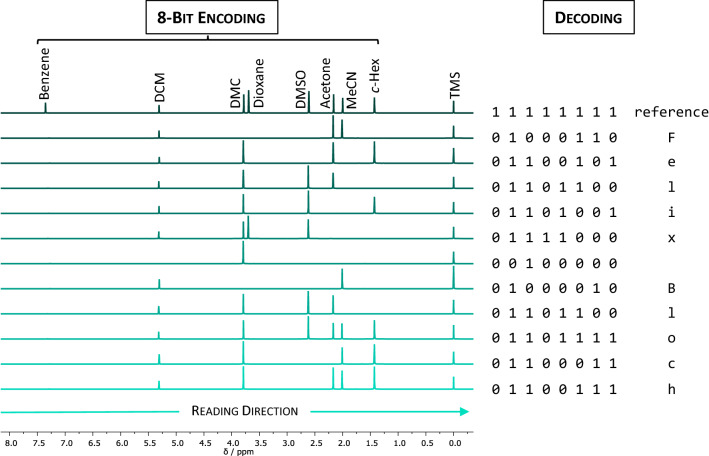
Encoding and decoding by ^1^H NMR analysis. “Felix␣Bloch”, who was awarded the Nobel Prize together with Edward Mills Purcell (Supplementary Fig. 1) in 1952^70,71^, was encoded and decoded in mixtures of up to eight information-containing compounds via an 8-bit ASCII code. The reading direction was specified from low to high field and the ordering via manual placement in the sample holder. The absence or presence of a compound signal in the spectra was retranslated to a sequences of “0”s and “1”s to reconstruct the binary code.

As a first and simple proof-of-concept, mixtures of up to nine different molecules, which each shows only one specific singlet ^1^H NMR-signal, were mixed in an NMR tube (Supplementary Table 1). Eight of them were used to encode an eight-bit (one byte) American Standard Code for Information Interchange (ASCII), whereas the last molecule (TMS) serves as a reference for the chemical shift. All of the information-containing chemicals are commercially available and standard solvents in a common laboratory. ASCII is a character encoding standard that allows 256 characters to be translated into binary code. These include not only the alphabet, but also numbers, punctuations, and special characters. The reading direction was defined from left to right within the ^1^H NMR spectrum, i.e., from low field to high field. For the later readout, a reference spectrum, a mixture that contained every of the eight information-containing compounds and thus the information of 11111111, was recorded (Fig. 1). To encode a certain character, the required molecules were added to write a “1” or left out for a “0” in binary language. An example is the letter “F” (in ASCII 01000110), which translates to DCM, acetone, MeCN, which were mixed with CDCl_3_ and the reference substance TMS to obtain the desired peak pattern (see Fig. 1 and Supplementary Table 1 for solvents and their order). In order to write a word, the sequence of the letters is determined by the manual placement of the eight-bit NMR tubes into the instrument sample holder in the correct order. Afterwards, the reading process works vice versa and is based on an alignment principle. The reference spectrum is compared to the individual eight-bit spectrum to be evaluated. Depending on the compound mixture, the obtained peaks are slightly shifted towards higher or lower ppm. The average peak maximum as well as the largest chemical shifts for a certain signal were determined in all measurements (Supplementary Table 1) and remained unproblematic for the read-out. With the presence of a signal within the standard deviation of the respective chemical shift, the value “1” is defined, otherwise a “0” is defined in case of absence. Thus, the NMR peak pattern is retranslated into the ASCII code and the associated character. Using this method, the names “Felix␣Bloch” (Fig. 1) and “Edward␣Mills␣Purcell” (Supplementary Fig. 1) were successfully encoded into 31 molecular mixtures (in total 248 bits) and decoded manually via NMR spectroscopy. Both were awarded the 1952 Nobel Prize in physics “for their development of new methods for nuclear magnetic precision measurements and discoveries in connection therewith”^70,71^.

### Molecular data storage using GC

To underline the simplicity and efficiency of this strategy of data storage in molecular mixtures, the writing and reading process was easily transferred to a standard GC-FID system. Here, we increased the storage capacity per mixture to 24 bits (3 bytes) by using 24 commercially available molecules, each of them with a different retention time in the chromatogram (see Supplementary Table 2 for the compound list and their order). Thus, in one mixture, three characters can be stored in a binary ASCII form (triads). *n*-Tetradecane was added to every mixture as the reference. Analogously to the NMR approach discussed above, a reference chromatogram of a mixture containing all molecules was recorded. By applying the from left-to-right reading (lower to higher retention time) and alignment strategy, the name of our university “Karlsruhe␣Institute␣of␣Technology” was successfully written and manually decoded using 11 mixtures (in total 264 bits, Supplementary Fig. 2). The order of the triads is also determined by placing the samples into the GC autosampler in the predefined order.

The challenge of sorting the information-containing molecules, whether it is sequence defined macromolecules or molecular mixtures, has been addressed by applying different approaches. Either by an “internal” position mass tag^47,58^ or a short ordering sequence^48^, or by the “external” arrangement of the samples on e.g. a surface^38,61,63^. We have so far shown the external arrangement of the samples for the data storage via NMR and GC, but we would also like to present a simple way for the internal approach. The reference substance *n*-tetradecane was therefore varied in its concentration in increments of 1 mg per sample and termed as the “ordering” compound in this context. Using this approach, only one more compound had to be added to the system, acting as the internal standard (2,6-dimethylphenol) to circumvent signal intensity deviations caused by e.g., variations of the injection volume or pipetting errors. Thus, the integral ratio of the ordering compound relative to the internal standard is calculated and the descending order of these values determines the sequence of the information pieces (Supplementary Fig. 4). This way, an internal sorting is achieved, and the information-containing samples can be stored and analyzed in any order, achieving always the correct original data.

**Figure 2:**
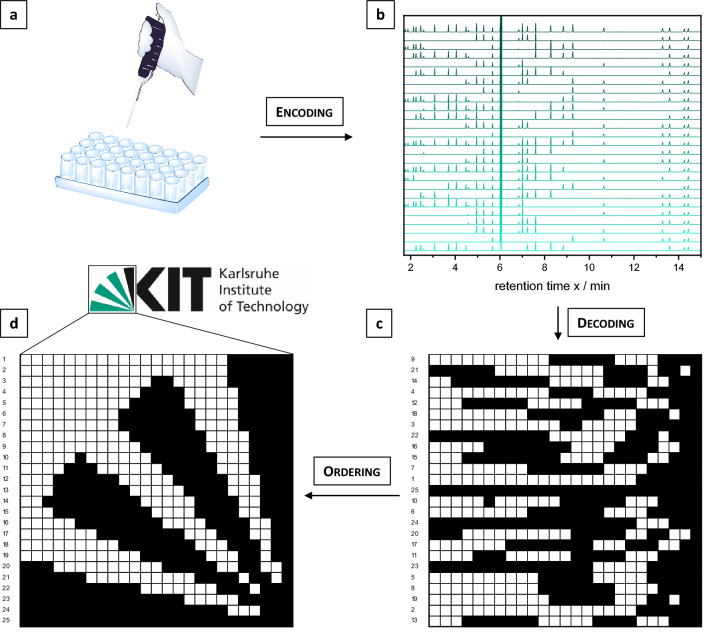
Schematic representation of bitmap coding. (**a**) Production of mixtures of up to 25 information-containing compounds per sample (25 bits) plus two reference molecules. (**b**) Randomly stacked, as obtained, GC chromatograms of information-containing samples plus reference chromatogram containing all compounds. (**c**) Translation of chromatograms into 25 × 25-pixel bitmap via alignment principle with the reference chromatogram. The absence of a signal is translated into a white pixel and the presence into a black pixel. The signals for the internal standard and the “ordering” compound were excluded from the translation process. (**d**) Ordering of the pixel lines according to the integral ratio of the two reference signals (Supplementary Fig. 4) revealing a picture of a fan, which symbolizes the fan shape of the city Karlsruhe and is also part of the KIT logo. The KIT logo was copied and modified with permission of the KIT. © Karlsruhe Institute of Technology.

For an illustration of the sorting process, a part of the KIT logo, which symbolizes the fan-shape of the city Karlsruhe, was saved as an image in a 25 by 25 bitmap by using 25 mixtures, containing 25 bits of information each (Fig. 2). If a black pixel is painted in the picture, the corresponding compound was added into the mixture to produce the required signal at that specific position in the data set. On the other hand, for a white pixel, the respective molecule was left out. The decoding process works vice versa again by comparison with a reference chromatogram. The presence of a compound and thus a signal stands for a black pixel and the absence of the molecule for a white one. In the schematic overview provided in Fig. 2, the information-containing mixtures were prepared in the first step (a) and analyzed in a random order. The unsorted chromatograms are depicted in (b) and were translated into the corresponding bitmap (c). At this point of the decoding process, the original information is not readable due to the disordering, which underlines the importance of the internal “ordering” compound. After the sorting process of the information pieces, the correct image was obtained (d). The used compounds are listed in Supplementary Table 2 and the 625 bitmap was manually written and decoded error-free. To optimize the read-out process in terms of reading speed and error-proneness, we next developed a decoding software, which is explained in detail in the following section.

### Computer assisted read-out of GC mixtures

In order to establish a faster and more efficient read-out of the data, different research groups have developed custom-made decoding software^47,58,69^. Here, we present a software tool for automatically decoding the data sets obtained by gas chromatography. First, some calculations were necessary to adjust the settings of the software, as will be explained in detail. Small deviations in the retention time of a certain molecule in GC measurements cannot be avoided. These retention time offsets were calculated manually for each signal with the data sets corresponding to the QR code and summarized in Supplementary Table 2 and visualized in Supplementary Fig. 3. The average retention time x_Ref_ of all used molecules was calculated via a three-fold determination of the reference sample measurement, and starting from this value, the distance to the maxima with the largest ± x-value shift over all 75 measurements (three-fold determination of each out of the 25 mixture) was determined. From these values, the width of the x-axis (ω) section was calculated, in which all maxima of the corresponding molecule are located. The largest deviation from the average retention time was observed for methyl stearate (Δ − x_MAX_ = 10.83 × 10^–3^ min.). In order to avoid errors in the decoding process, a higher value (Δ ± x_MAX_ = 15.0 × 10^–3^ min.) was defined in the settings of the software to make it more robust against major deviations. Thus, a width of ω = 30.0 × 10^–3^ min. is set as x-range, in which it searches for a maximum in information-containing molecule mixtures. These small deviations did not influence a manual or automated read-out. Furthermore, a y-threshold of y = 50 mV was set to eliminate the baseline noise. The integration area for the ordering compound signal, *n*-tetradecane, was defined as [x_1_ = 5.98 min.; x_2_ = 6.10 min.] and for the internal standard, 2,6-dimethylphenol, as [x_3_ = 5.63 min.; x_4_ = 5.70 min.].

**Figure 3:**
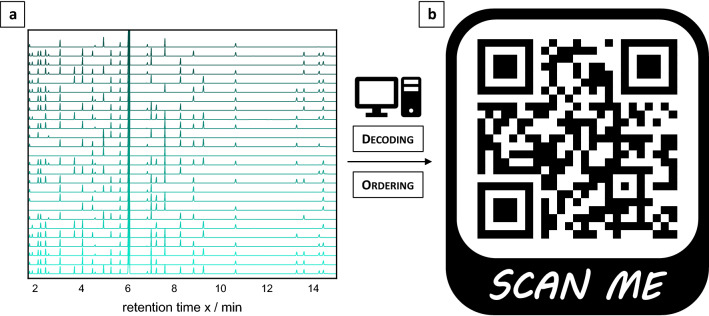
Schematic representation of QR coding. (**a**) Randomly ordered GC chromatograms of information-containing samples plus reference chromatogram containing all compounds. (**b**) Bitmap of a 25 × 25-pixel QR code containing 625 bits of information. Encoding was achieved via GC in 25 mixtures using 25 information compounds (25 bits) plus two reference molecules and a reference sample containing all molecules. Decoding was performed manually and using a new decoding software. The QR leads to the homepage of the KIT (https://www.kit.edu/index.php).

In the first step, the CSV (Comma-Separated Values) data files obtained from the GC instrument were imported into the script. For the ordering process, the reference signals were integrated using the trapezoidal rule. The values obtained for the *n*-tetradecane signal are divided by the ones for the internal standard (2,6-dimethylphenol). These ratios are then arranged in ascending order, defining the sequence of the information-containing molecule mixtures (Supplementary Fig. 4). Then, the software calculates the absolute maxima of each data set by comparing each y-value with its nearest neighbor in ± x direction and recognizes the reference sample based on the presence of the highest amount of found maxima. In the last step, the x-values of the maxima of the reference chromatogram are compared with those of the individual mixtures within the specified tolerance of ω = 30.0 × 10^–3^ min. The reference signals were excluded from this step, as they do not carry information, apart from the sample order already evaluated above. If a match and thus the presence of a compound is determined, a black pixel is displayed. On the other hand, if a maximum is not observed and thus the absence of a compound is determined, a white pixel is displayed. With help of this software, a QR code (Fig. 3), referring to the homepage of the Karlsruhe Institute of Technology, could be decoded with 100% accordance. To confirm the errorless functioning of the software, the image of the “fan” was re-read automatically with the same precision. The individual steps of the entire encoding and decoding process is shown in the flowchart in Supplementary Fig. 5.

In summary, we presented a fast and efficient strategy for data storage using commercially available chemicals. Mixtures of up to 25 information-containing compounds were prepared manually and decoded via spectroscopic (^1^H NMR) or chromatographic (GC) approaches. Thus, the writing and reading of binary ASCII codes and bitmaps was shown as well as an easy ordering approach. We developed a decoding software, which automatically put the data sets into correct order and guaranteed a faster read-out of the original information. Thus, we have introduced a simple strategy for molecular data storage that avoids complicated syntheses and complex analytical methods by demonstrating encoding and automated decoding of QR codes. Especially the use of a standard GC-FID instrument for the read-out cheapens the analysis by more than one order of magnitude in terms of acquisition cost, if compared to the typically available NMR or MS equipment.

## Methods

### Materials

1,2-Propanediol (Acros Organics, ACS reagent), 1,10-decanediol (Acros Organics, 99%), 1,12-dodecanediol (Sigma Aldrich, 99%), 1,4-diethoxybenzene (TCI, > 98.0%), 1,8,9-trihydroxyanthracene (Alfa Aesar, 97%), 1-adamantanol (Acros Organics, 99%), 1-hexanol (Sigma Aldrich, 98%), 2,3-butanediol (Sigma Aldrich, 98%), 2,6-dimethoxyphenol (Sigma Aldrich, 99%), 2,6-di-^*t*^Bu-4-methylphenol (Sigma Aldrich, ≥ 99.0% (GC)), 2-naphthaleneethanol (Sigma Aldrich, 98%), 2-phenylethanol (Sigma Aldrich, ≥ 99.0% (GC)), 3,3’,5,5’-tetramethylbiphenyl (Alfa Aesar, 97 + %) 4-ethylphenol (Sigma Aldrich, 99%), 4-methoxyphenol (Sigma Aldrich, 99%), 9-anthracenemethanol (Sigma Aldrich, 97%), acetone (Honeywell, ≥ 99.8%, for HPLC), acetonitrile (MeCN, Fisher Scientific, HPLC Gradient grade), benzene (Sigma Aldrich, anhydrous, 99.8%), benzyl alcohol (Honeywell, ≥ 99.0%), chloroform-*d* (CDCl_3_, Eurisotop®, 99.80 atom-% D, stabilized with silver foil), cyclohexane (VWR, HPLC grade), cyclohexanol (Sigma Aldrich, 99%), cyclooctane (Fluka, ≥ 99.0% (GC)), dichloromethane (DCM, Fisher Scientific, ≥ 99.8%, HPLC grade), diethylene glycol (Sigma Aldrich, ≥ 99.0% (GC)), dimethyl carbonate (DMC, Acros Organics, 99%), dimethyl sulfoxide (DMSO, Fisher Scientific, ≥ 99.9%), dioxane (Acros Organics, 99 + %, extra pure, stabilized), ethyl acetate (VWR, HPLC grade), *n*-hexadecane (Alfa Aesar, 99%), methyl oleate (ABCR, 96%), methyl stearate (Acros Organics, mixtures of homologs), *n*-tetradecane (Sigma Aldrich, ≥ 99.0% (GC)), tetraethylene glycol monomethyl ether (TCI, > 98.0%), tetramethyl silane (TMS, ABCR, 99.9%, NMR grade), triethylene glycol (Sigma Aldrich, 99%).

### Instrumentation

#### Nuclear magnetic resonance (NMR) spectroscopy

NMR spectra were recorded on a Bruker AVANCE DPX spectrometer operating at 400 MHz for ^1^H measurements with 16 scans, a delay time *d*_1_ of 1 s, and an acquisition time of 4 s at 298 K. CDCl_3_ was used as solvent and the respective resonance signal of TMS at 0.00 ppm served as reference for the chemical shift δ / ppm. For the preparation of the mixtures, 10 µL of the respective analyte was dissolved in 500 µL CDCl_3_.

#### Gas chromatography (GC)

GC measurements were performed using an Agilent 8860 gas chromatograph with a HP-5 column (30 m × 0.32 mm × 0.25 µm) and a flame ionization detector (FID). The measurements were carried out using the following heating program of the oven: initial temperature 95 °C, hold for 1 min, ramp up to 200 °C with a rate of 15 °C·min^−1^, hold 200 °C for 4 min, ramp up to 300 °C with a rate of 15 °C·min^−1^ and then holds 300 °C for 2 min. The samples were prepared as followed: Stock solutions with a concentration of *c* = 50 mg·mL^−1^ were prepared in EA. For 1-adamantanol: *c* = 25 mg·mL^−1^, 1,10-decanediol and 9-anthracenemethanol: *c* = 12.5 mg·mL^−1^, 1,12-dodecanediol and 1,8,9-trihydroxyanthracene: *c* = 8.33 mg·mL^−1^, due to solubility issues. The respective volumes to achieve 1.5 mg of substance were added to the mixture. 900 µL of the internal standard (*c* = 1.5 mg·mL^−1^ in EA) was added. The second reference, *n*-tetradecane, was added in 1 mg increments, starting from 1 mg for the first mixture and 26 mg for mixture number 26. All samples were filtered by syringe filter prior to use, to avoid plugging of the injection setup or the column. The injection volume was set to 1 µL and the injection temperature to 220 °C.

## Supplementary Information

Supplementary Information.

## Data Availability

All relevant data is included as supplementary information and is available from the corresponding author upon request.

## Code availability

The relevant software is included as Supplementary Software 1. A description, explaining the code is provided in Supplementary Data 1. Data sets for demonstrating the function of the software is provided in Supplementary Data 2.
