# Supplementary material for: Molecular data storage with zero synthetic effort and simple read-out
Source: Sci Rep. 2022 Aug 16;12:13878. doi: 10.1038/s41598-022-18108-9 (PMC9381582; doi:10.1038/s41598-022-18108-9)
Supplement: Supplementary file 1 — Supplementary Information. [file 41598_2022_18108_MOESM1_ESM.pdf]

Supplementary Information

Molecular data storage with zero synthetic effort and simple read-out

Philipp Bohn,[1] Maximilian P. Weisel,[1] Jonas Wolfs,[1] Michael A. R. Meier[1,2]*

[1] Laboratory of Applied Chemistry, Institute of Organic Chemistry (IOC), Karlsruhe Institute of Technology (KIT), Straße am Forum 7, 76131 Karlsruhe, Germany, E-mail: m.a.r.meier@kit.edu. Homepage: http://www.meier-michael.com.

[2] Institute of Biological and Chemical Systems – Functional Molecular Systems (IBCS-FMS), Karlsruhe Institute of Technology (KIT), Hermann-von-Helmholtz-Platz 1, 76344 Eggenstein-Leopoldshafen, Germany.

Supplementary Table 1 Chemical shifts of molecules used for data storage in NMR	
**compound**	**Average chemical shift and maximal deviation δ / ppm**
Benzene	7.3546
DCM	5.3084 ± 0.0092
DMC	3.7861 ± 0.0044
Dioxane	3.6972 ± 0.0038
DMSO	2.6137 ± 0.0055
Acetone	2.1666 ± 0.0038
MeCN	2.0052 ± 0.0060
Cyclohexane	1.4278 ± 0.0007
TMS	0.0000 ± 0.0000

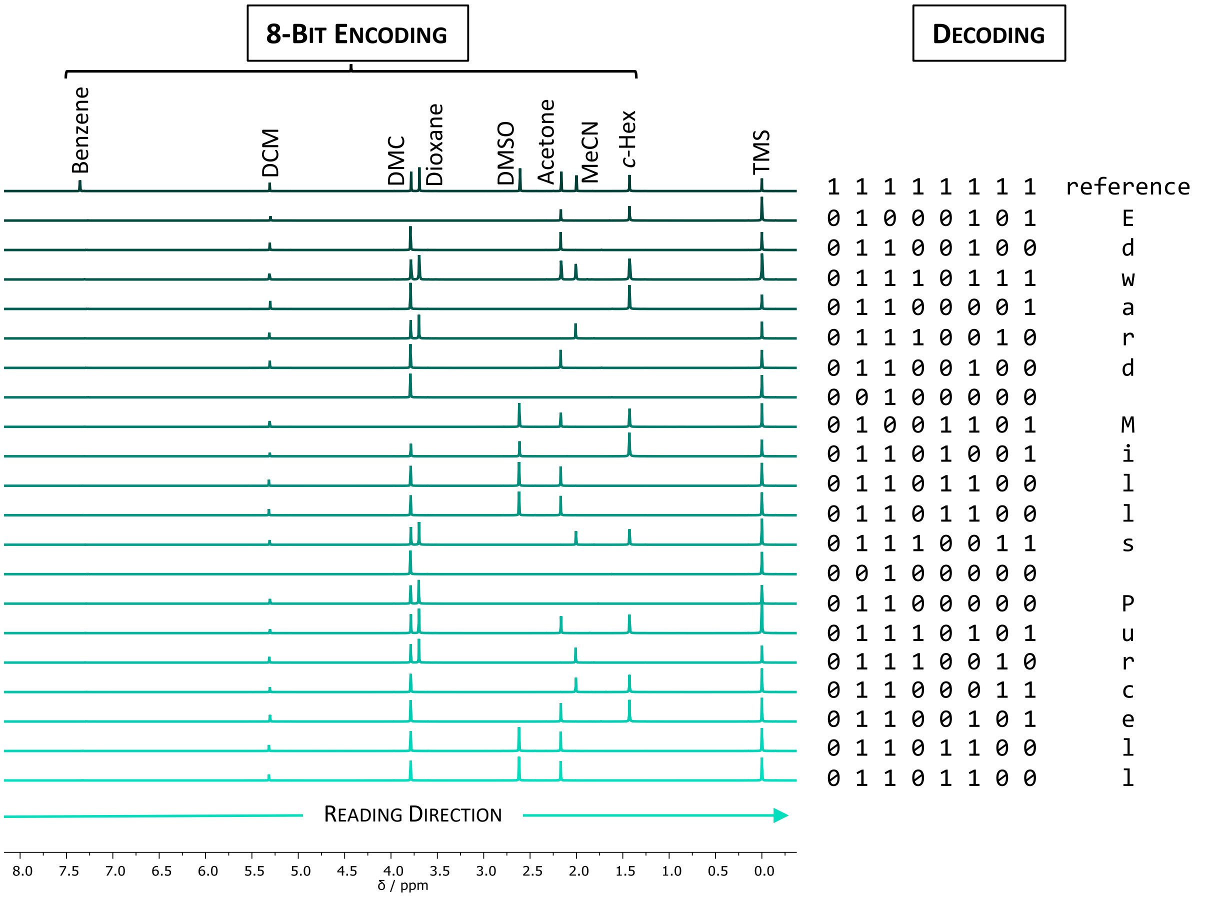

Supplementary Figure 1 Encoding and decoding with ^{1}H NMR analysis. "Edward␣Mills␣Purcell", who was awarded the Nobel Prize together with Felix Bloch in 1952,[1,2] was encoded and decoded in mixtures of up to eight compounds *via* an 8-bit ASCII code. The reading direction was specified from low to high field and the ordering *via* manual placement in the sample holder. The absence or presence of a compound signal in the spectra was retranslated in the binary code into "0" and "1".

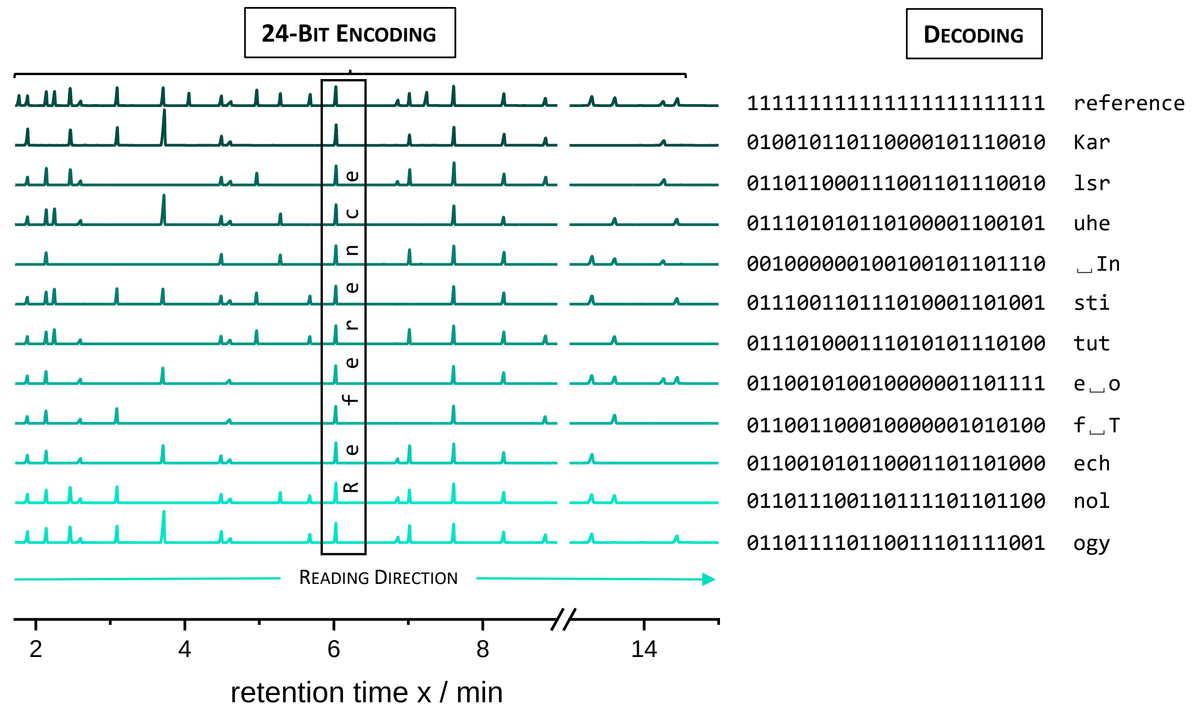

Supplementary Figure 2 Encoding and decoding with GC analysis. “Karlsruhe␣Institute␣of␣Technology” was encoded and decoded in mixtures of up to 24 compounds *via* an 8-bit ASCII code (3 byte per mixture). The reading direction was specified from lower to higher retention time and the ordering *via* manual placement in the sample holder. The absence or presence of a compound signal in the chromatogram was retranslated in the binary code into “0” and “1”.

**Supplementary Table 2 Retention time and processed data of molecules used for data storage in GC**				
**Compound**	**x_{Ref}[a] /min**	**Δ-x_{MAX} × 10^{-3} [b]**	**Δ+x_{MAX} × 10^{-3} [c]**	**ω × 10^{-3} [d]**
1,2-Propandiol	1.768	2.920	1.250	4.170
2,3-Butandiol	1.882	2.505	0.835	3.340
1-Hexanol	2.135	3.335	0.835	4.170
Cyclohexanol	2.245	2.505	0.835	3.340
Cyclooctane	2.456	1.665	2.505	4.170
Diethylenglycol	2.590	2.920	1.250	4.170
Benzyl alcohol	3.077	2.230	2.160	4.390
2-Phenylethanol	3.698	2.495	2.505	5.000
4-Ethylphenol	4.049	3.330	1.670	5.000
4-Methoxyphenol	4.483	4.165	1.665	5.830
Triethylenglycol	4.599	10.00	1.670	11.670
1-Adamantanol	4.958	6.250	1.250	7.500
1,4-Diethoxybenzene	5.273	2.923	1.835	4.758
2,6-Dimethylphenol	5.672	2.920	2.080	5.000
TEGMeO	6.850	4.580	2.920	7.500
2,6-Di-^{t}Bu-4-methylphenol	7.012	4.995	1.665	6.660
1,10-Decandiol	7.234	4.170	5.000	9.170
n-Hexadecane	7.601	2.910	2.090	5.000
2-Naphthaleneethanol	8.275	2.080	1.250	3.330
1,12-Dodecandiol	8.844	8.750	1.250	10.000
3,3’,5,5’-Tetramethylbiphenyl[e]	9.265	6.665	1.665	8.330
Methyl palmitate[e]	10.653	5.420	2.920	5.712
Methyl oleate	13.284	1.670	5.000	6.670
Methyl Stearate	13.599	10.830	2.500	13.330
1,8,9-Trihydroxyanthracene	14.248	3.330	4.170	7.500
9-Anthracenemethanol	14.437	5.830	3.330	9.160

Values were determined from the data sets measured for the encoding of the QR code; [a] averaged x-values (x_{Ref}) of the respective peak maxima (retention time) of the associated compound calculated from the reference spectra (three-fold determination); [b] distance to the maxima over all measurements with the largest Δx-value in the direction of lower (Δ-x_{MAX}) and [c] higher (Δ+x_{MAX}) retention times; [d] range in which all maxima of the respective compound are located ($\omega = \Delta+x_{MAX} + \Delta-x_{MAX}$); [e] compound was not used for the data storage shown in Supplementary Figure 2.

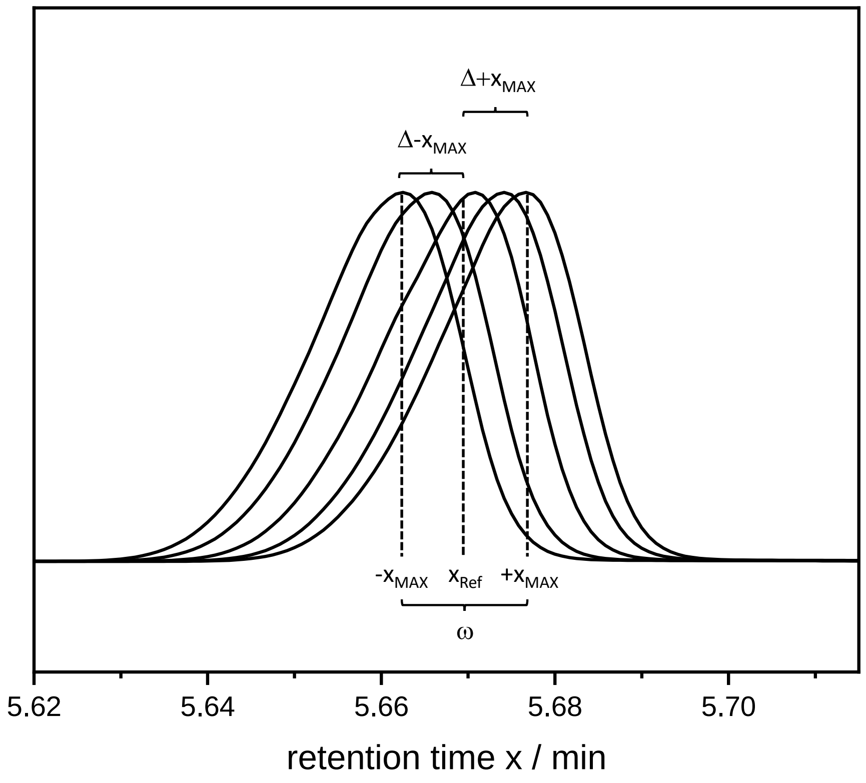

Supplementary Figure 3 Schematic representation of the calculated values in Supplementary Table 2.

$x_{Ref} = (-X_{MAX}) + (+X_{MAX})/2$; $\Delta - x_{MAX} = x_{Ref} - (-x_{MAX})$; $\Delta + x_{MAX} = (+x_{MAX}) - x_{Ref}$;

$\omega = (-X_{MAX}) + (+X_{MAX})$

Note that the + and – sign direct before X_{MAX} is no arithmetic operator.

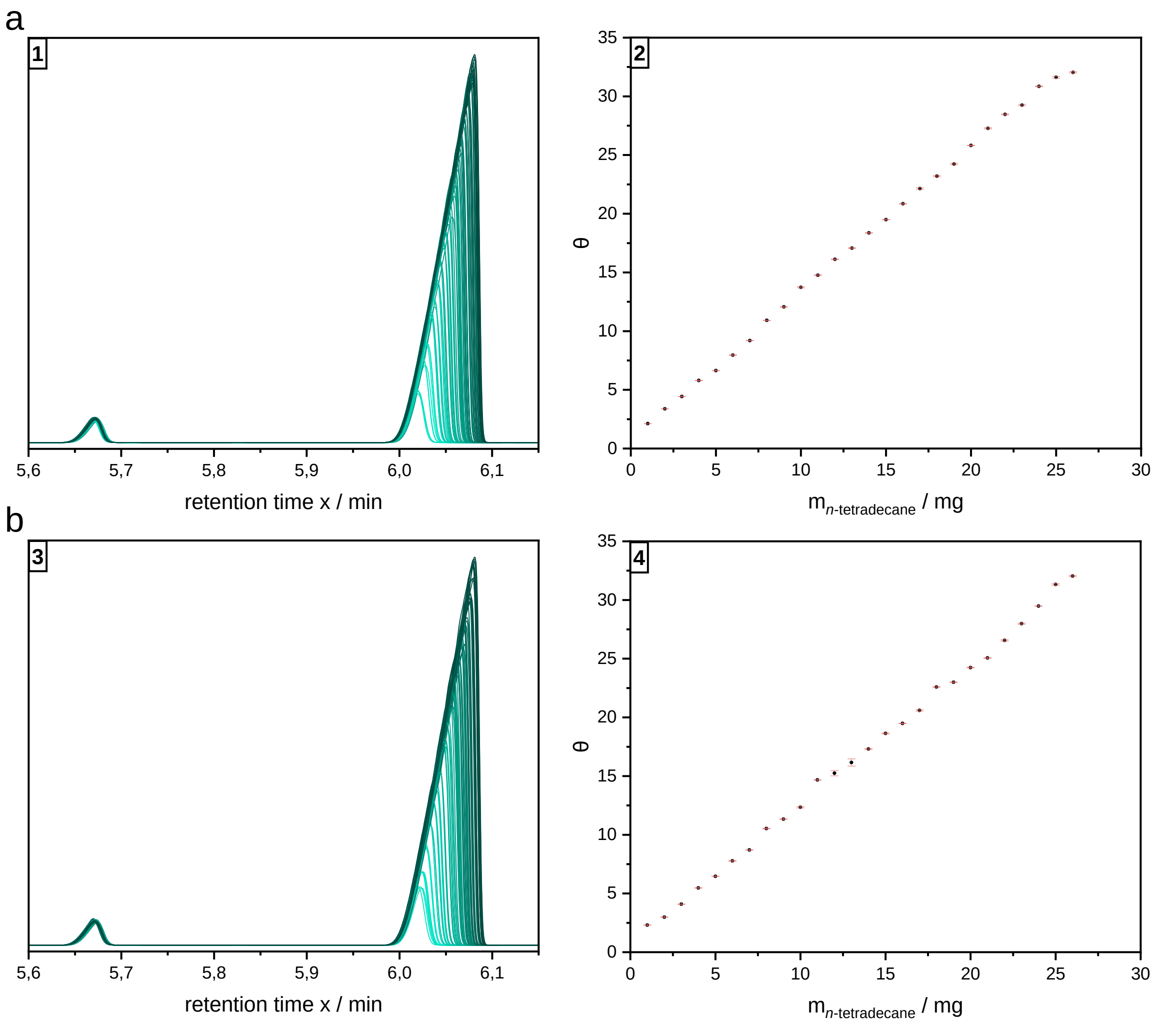

Supplementary Figure 4 Calibration curve and chromatograms of the reference. Ordering of data sets dependent on the integral ratio of the two reference signals of *n*-tetradecane $[x_1 = 5.98;\ x_2 = 6.10]$ and 2,6-dimethylphenol $[x_3 = 5.63;\ x_4 = 5.70]$ calculated by a three-fold determination $(f(x), g(x), h(x))$. **a** represent the data set for the “fan” bit map and **b** for the QR code. **1** and **3** Cutout of GC chromatograms focusing on the two reference peaks. Left signal: 2,6-dimethylphenol with same concentration in each mixture. Right signal: *n*-tetradecane with varying concentration. **2** and **4** Plot of the averaged integral ratio θ against the amount of *n*-tetradecane added to the respective mixture with $\theta = \overline{\mathrm{X}}\left[\int_{x_1}^{x_2} f(x); \int_{x_1}^{x_2} g(x); \int_{x_1}^{x_2} h(x)\right] \times \overline{\mathrm{X}}\left[\int_{x_3}^{x_4} f(x); \int_{x_3}^{x_4} g(x); \int_{x_3}^{x_4} h(x)\right]^{-1}$.

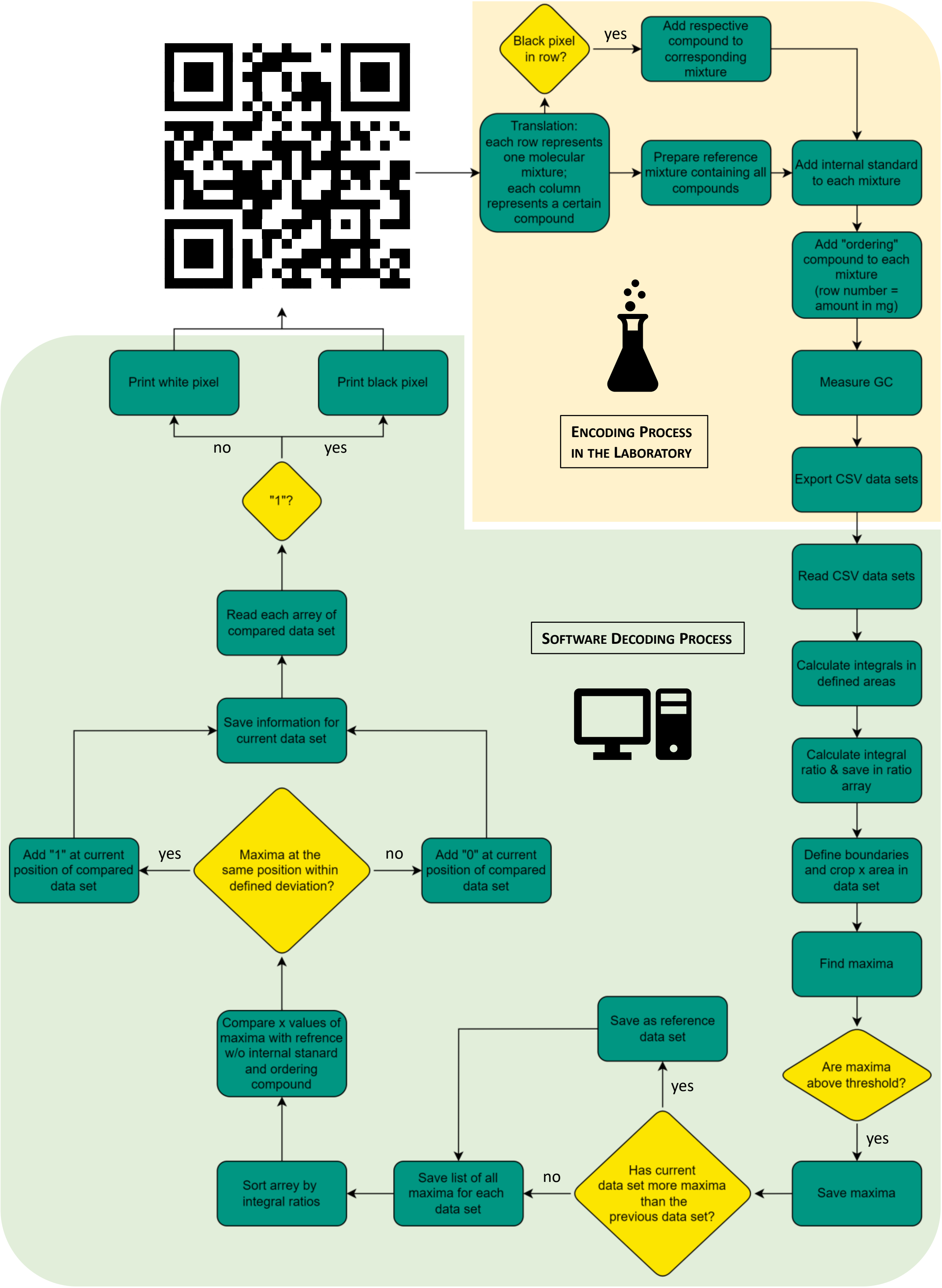

Supplementary Figure 5 Flowchart describing the encoding and decoding process for the QR code. The individual steps for the encoding process in the laboratory are highlighted in light yellow and for the decoding process *via* the software in light green. The processing steps are represented in green rectangles and the decision points in yellow rhombi. The QR leads to the homepage of the KIT (https://www.kit.edu/index.php). For detailed information about the code, refer to the provided files (README.txt and the read.py).

References

1. Bloch, F. Nuclear Induction. *Phys. Rev.* **70,** 460–474; 10.1103/PhysRev.70.460 (1946).
2. Purcell, E. M., Torrey, H. C. & Pound, R. V. Resonance Absorption by Nuclear Magnetic Moments in a Solid. *Phys. Rev.* **69,** 37–38; 10.1103/PhysRev.69.37 (1946).
